# Scotland's Care for Sanitary Science

**Published:** 1921-08-13

**Authors:** 


### Scotland's Care for Sanitary Science.

The "Transactions of the Incorporated Sanitary Association of Scotland, 1920," recently published, show that our colleagues north of the Tweed are well in the van of sanitary science. We have a further assurance of this in the remarks of Bailie Kelly at the opening of the annual Congress when he stated, "Everyone knows that Scotland is the pioneer of sanitary arrangements throughout the world. I am of opinion that the Incorporated Sanitary Association of Scotland will be able to keep well ahead of any other country in the world so far as sanitary matters are concerned." Certainly the excellent papers read at the Congress and the high level of the discussions which followed their reading warrant Bailie Kelly in some optimism. The Congress began by a popular lecture on health problems by Professor Glaister. In this he dealt with many aspects of sanitation, and one of his best references was in defence of that much-maligned and misunderstood statistician, Malthus. Indeed, after we had read this we made up our minds to take an early opportunity of reading his original work, for it seems to us that in regard to this author—and the remark will apply generally—we are much too prone to form opinions from the excerpts of critics and admirers instead of consulting the original work. This paper was followed by the Presidential address by Provost John Raffan, J.P., of Stirling. The subject was "Towards the Ideal City" (Retrospect, Aspect, and Prospect). In this he dealt with the evils and remedies of urbanisation, and, judging by one photograph of a "Typical Scottish Wynd," it would seem that in Scotland they have a long road to travel before they arrive at even a tolerable condition, much less an ideal city.

One of the most practical addresses was given by Mr. John Fyfe, a sanitary inspector of Stirling, on the "Sanitary Condition of Picture Houses," and if he laid a little too much stress on the percentage of $CO_2$ in the air of these places, he demonstrated at least that he had made a thorough study of his subject and succeeded in arousing the interest of the Scottish Board of Health to undertake a further investigation on somewhat broader lines. The housing question and the milk supply came in for investigation and criticism, and the Congress ended with two excellent papers on "Some Reasons why we fall so far short of being an A1 Nation," by Dr. Ernest Watt, Scottish Board of Health, and "Smoke Prevention in Relation to Health," by ex-Bailie Wm. B. Smith, O.B.E. There is one sentence on the former's paper we cannot refrain from quoting, as it deserves world-wide publicity, "As it is a woman's whole work to attend to a young child, and do the necessary housework, we must keep in mind the fact that work outside the home is incompatible with efficient motherhood. In fact, so long as our social economy makes employment of married women necessary, we shall find the effects of neglect upon the children."

